# Generation time, life history and the substitution rate of neutral mutations

**DOI:** 10.1098/rsbl.2014.0801

**Published:** 2014-11

**Authors:** Jussi Lehtonen, Robert Lanfear

**Affiliations:** 1 Evolutionary Biology, Zoological Institute, University of Basel, Vesalgasse 1, 4051 Basel, Switzerland; 2 Centre of Excellence in Biological Interactions and Division of Evolution Ecology and Genetics, Research School of Biology, The Australian National University, Canberra, Australian Capital Territory 0200, Australia; 3 National Evolutionary Synthesis Center, Durham, NC 27705-4667, USA; 4 Department of Biological Sciences, Macquarie University, Sydney, New South Wales 2109, Australia

**Keywords:** molecular evolution, neutral substitution rate, life history, generation time, fecundity

## Abstract

Our understanding of molecular evolution is hampered by a lack of quantitative predictions about how life-history (LH) traits should correlate with substitution rates. Comparative studies have shown that neutral substitution rates vary substantially between species, and evidence shows that much of this diversity is associated with variation in LH traits. However, while these studies often agree, some unexplained and contradictory results have emerged. Explaining these results is difficult without a clear theoretical understanding of the problem. In this study, we derive predictions for the relationships between LH traits and substitution rates in iteroparous species by using demographic theory to relate commonly measured life-history traits to genetic generation time, and by implication to neutral substitution rates. This provides some surprisingly simple explanations for otherwise confusing patterns, such as the association between fecundity and substitution rates. The same framework can be applied to more complex life histories if full life-tables are available.

## 1. Introduction

Understanding variation in rates and patterns of molecular evolution is an important part of modern biology. In molecular evolution, generation time (*T*) is expected to be one of the primary determinants of the substitution rate of neutral mutations per time-unit, because species with short *T* copy their genomes more often, accruing more copy errors per year [1,2]. Although this reasoning is simple, the link between life history (LH) and *T* is less obvious. For semelparous species, age at first reproduction (*b*) accurately represents *T*, because all of the offspring are produced in the first reproductive event. However, for iteroparous species, *T* cannot be fully described by age at first reproduction, because reproductive events may continue to occur long after the first event.

The most general measure of *T*, covering semelparity and iteroparity, is the mean age of the parents of a set of new-born individuals (table 1) [3,4]. This defines the average time between reproductive events in a lineage, and the timeframe for the accumulation of errors generated during such events. Theoretical work [5] shows that this definition of *T* scales the mutation rate per generation (*U*) to the neutral substitution rate (*K*) per time unit with a simple equation1.1
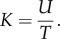
Note that *U* by itself determines the probability of a new mutation appearing in an offspring [4,6], and therefore lacks a timescale. Because *T* in iteroparous species is determined by LH-traits such as fecundity and survival, the same must apply to *K*, independently of variation in *U*.

**Table 1.** Notation, definitions and demographic equations used in the model.

notation	name of parameter, variable or equation	definition
*K*	neutral substitution rate	rate at which mutations with no effect on fitness are fixed in the genome per time unit
*U*	mutation probability per generation	the probability that, at a focal site, an offspring has a mutation that its parents do not have
*r*	population intrinsic growth rate (Malthusian parameter)	the exponential *per capita* rate of population increase
*f*(*x*)	fecundity	rate of reproduction in female offspring per mother (age *x*) per year
*b*	age at first reproduction	age at which individuals reproduce for the first time
*c*	length of reproductive time window	time in years during which an individual is reproductively active, if it does not die for stochastic reasons earlier
*d* (=*b* + *c*)	age at last reproduction	age in years at which an individual is potentially able to reproduce for the last time, if it has not died for stochastic reasons earlier (close to maximum lifespan in species without menopause)
*σ*	offspring survival probability	proportion of new-born offspring surviving to reproductive age
*μ*	adult mortality rate	instantaneous mortality rate of reproductive individuals
*l*(*x*)	survival probability	probability of survival from birth to age *x,* taking into account both juvenile and adult survival
*σf*	recruitment	rate of offspring surviving to maturity per mother per year (i.e. juvenile survival multiplied by fecundity)
*T*	generation time	mean age of parents, averaged over new-born individuals in a population: 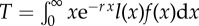
	Euler–Lotka equation	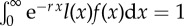

Comparative studies of molecular evolution typically compare estimates of LH-traits with *K*. The set of traits included in these studies varies, as does the definition that is used for *T*; for example, age at first reproduction has been interpreted as *T* in several studies [1,7–10]. However, these simple proxies can fail to account for important sources of variation in *T*, leading to potentially spurious correlations between *K* and LH-traits. For example, a correlation between rates of molecular evolution and fecundity was found in mammals [9], even after accounting for variation in other LH-traits. This has been interpreted as evidence for an association between fecundity and the neutral substitution rate, which is independent of generation time, and potential explanations have focused on mechanistic links between fecundity and mutation rates [9,11,12]. However, these hypotheses are not well supported by the available data, and the pattern still lacks a convincing explanation [11]. Furthermore, while one study ruled out a strong association of *K* with longevity [9], another found the opposite [13], but the latter did not include fecundity as a potential covariate. We suggest that these unexplained results can be partially resolved by properly accounting for LH-effects on *T*.

## 2. The model

The equation for *T* (table 1; [3,4]) requires, in principle, a full description of LH, which is usually not available for all species in comparative studies of molecular evolution. We therefore seek guidelines to predicting and understanding the effect of LH-traits on *T* and *K* in iteroparous species, in the absence of detailed data. We use the Euler–Lotka equation [4,14] (see table 1) to eliminate an unknown LH-parameter from the equation for *T*. The result is a predicted relationship between the remaining LH-traits and *T*, and by implication, *K*.

We assume that individuals survive to age of first reproduction (*b*) with probability *σ*, and thereafter have constant rates of mortality (*μ*) and reproduction (*f*) until age *d*. If they reach *d*, reproduction stops (death may or may not occur at the same time). Age-independent (after first reproduction) *μ* and *f* are reasonably realistic for many species [15,16]. In the electronic supplementary material, we show that the main results apply qualitatively even if some of these assumptions are relaxed (a further implicit assumption is that males and females have similar LHs and mutation rates; see [4,5] for methods to account for sex-specificity).

These assumptions imply that for ages *b* ≤ *x* ≤ *d* survival is *l*(*x*) = *σ*e^−*μ*(*x*−*b*)^, and the reproduction rate is
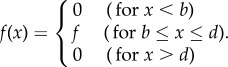
Substituting into the Euler–Lotka equation yields2.1
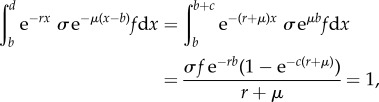
where *c* is the length of the reproductive time window (i.e. *d* = *b* + *c*). Now, recruitment can be solved as a function of adult mortality, and vice versa2.2a
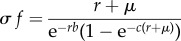
and2.2b
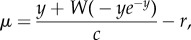
where *y* = *σfc*e^−*rb*^ and *W* is the principal branch of the Lambert W function [17] (see the electronic supplementary material for details). We then use (2.2*a*,*b*) to eliminate either recruitment or mortality from *T*
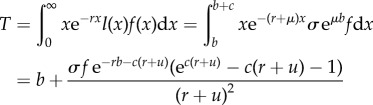
2.3a
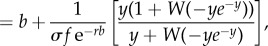
2.3b
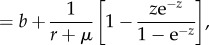
2.3c
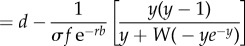
2.3d
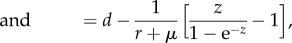
where *y* = *σf*e^−*rb*^*c* and *z* = *c*(*r* + *μ*).

Equations (2.3*a*,*b*) show how *T* deviates from its lower limit *b,* whereas (2.3*c*,*d*) show the deviation from the upper limit *d.* Our interest lies particularly in (2.3*a*), but the other forms are shown for completeness, and for comparison with previously used approximations of *T* as a function of adult survival and population growth [18,19].

**Figure 1.**
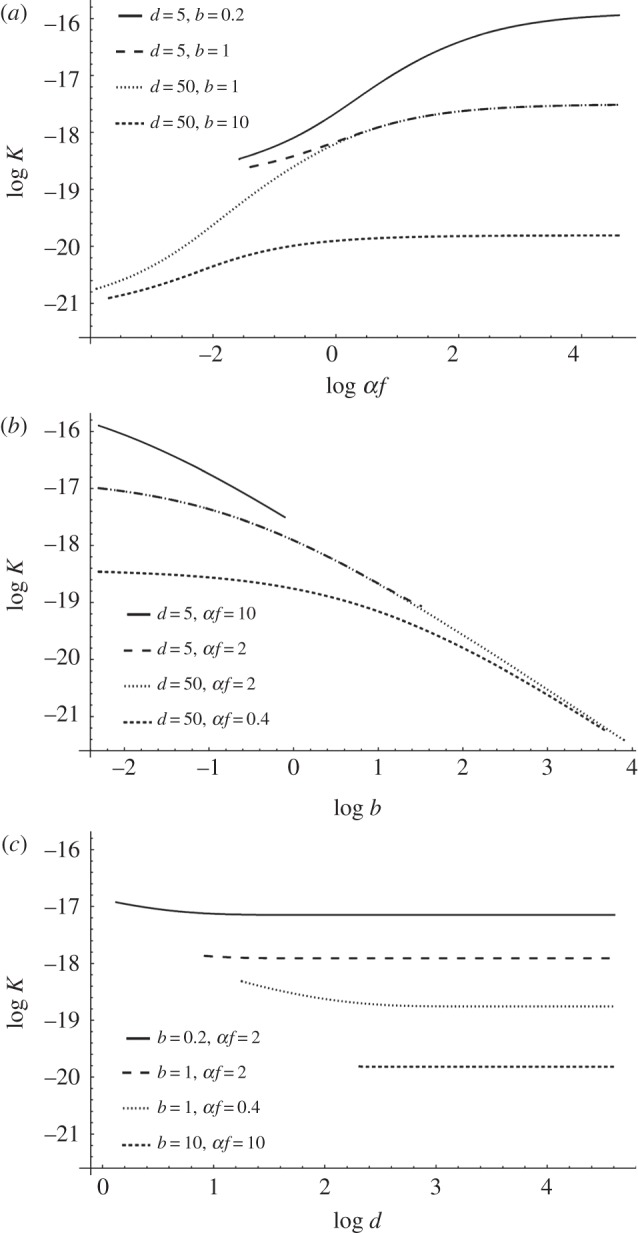
Neutral substitution rate as a function of (*a*) recruitment, (*b*) age at first reproduction and (*c*) age at last reproduction, when the other two variables are held constant. The figure is based on equation (2.3*a*), with constant population size (*r* = 0), *K* = *U*/*T* and *U* = 2.5 × 10^−8^ [20]. The direct causal effect of age at last reproduction on the substitution rate, as predicted by our model, is very small for most parameter combinations (panel (*c*), but see main text). Not all parameter combinations are possible (e.g. the constraints *b* < *d* and *σf*(*d* – *b*) > 1 must be fulfilled), which is why some curves are truncated. Displayed combinations illustrate the overall effect of LH-traits on *K*, but some sections of the curves may be unrealistic, even if possible in principle (e.g. early maturation combined with very low fecundity is unlikely in mammals).

Examining particular special cases of (2.3*a*) makes interpretation easier. If we assume that *r* → 0 and *c* → ∞ (no population growth or effect of senescence on *T*, reasonable first approximations in long-term evolution under natural conditions), (2.3*a*) yields2.4
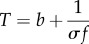
implying2.5
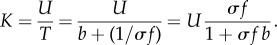
Equation (2.5) is an increasing, but saturating function of recruitment *σf*, compatible with the unexplained empirical observation that *K* increases with *f* [9]. The full equation (2.3*a*) can be seen as (2.4), corrected for maximum lifespan and population growth. Using (2.3*a*), we can plot estimates of the effect of each LH parameter on the neutral substitution rate (figure 1).

Here, we have used a continuous derivation, as a discrete model does not permit analytical solutions with a limited lifespan (*d*). However, equation (2.4) is similar if derived with a discrete model.

## 3. Results and discussion

Equations (2.3) and (2.4) make quantitative predictions for genetic generation time (*T*) from limited LH data. Because theory [4,5] predicts *T* to be an important component of neutral substitution rates (*K*) in many taxa, these results may both aid future studies of molecular evolution and clarify past studies. For example, our results suggest a simple explanation for the observed correlations between fecundity (*f*) and *K* [9]: all else being equal, an iteroparous population with high recruitment (*σf*) must have high adult mortality, short *T* (equation (2.4)) and high *K* (equation (2.5) and figure 1*a*). More generally, our results show that while recruitment and age at first reproduction (*b*) strongly influence *K* (via *T*), longevity (*d*) is predicted to have a very minor effect (figure 1*c* and the electronic supplementary material). These predictions are broadly in agreement with published comparative studies [1,9,11,21].

A separate question is whether LH traits are correlated with each other. Longevity should evolve in response to traits such as fecundity and age at first reproduction [22] and can also reflect extrinsic mortality [23]. Therefore, longevity alone could still be a reasonable predictor of substitution rates (as found by Lartillot & Delsuc [13]), simply because it is an evolutionary outcome of the same traits that determine *T*. Combined with the fact that fecundity was not included in [13], this could explain the discrepancies between seemingly similar comparative studies [9,13]. In general, comparative analyses should take into account the possibility that LH traits may evolve in syndromes and are known to often be correlated with each other [11].

The extent to which factors other than *T* affect *K* likely varies between taxa and genomes [11,21], potentially making the association between *T* and *K* harder to detect empirically. Natural selection may reduce the somatic mitochondrial mutation rate in long-lived species, producing evolutionary correlations between LH and *U* not accounted for by our model [24–26]. The link between *T* and *K* is also likely to be weakened if germline mutations accumulate throughout the lifespan of the organism, such as occurs in plants [21,27,28] and male mammals [29,30]. Nevertheless, our results have explanatory power to understand substitution rate variation as long as *U* has a component that does not accumulate continuously throughout an organism's lifetime (e.g. mutations that accumulate during meiosis in each generation; [9]).

Equations (2.1)–(2.5) assume repeated reproduction, and therefore apply only to iteroparous species (e.g. mammals, as studied in references [9,13]). However, equation (1.1) also applies to semelparous species, for which *T* is simply equal to *b*, as no further reproduction takes place after the first reproductive event. With semelparity, recruitment (*σf*) is therefore not expected to affect *T* or *K* via the processes described here, which may provide a way to test the theory with data.

## Supplementary Material

Supplementary material
