# Supplementary material for: Generation time, life history and the substitution rate of neutral mutations
Source: Biol Lett. 2014 Nov;10(11):20140801. doi: 10.1098/rsbl.2014.0801 (PMC4261869; doi:10.1098/rsbl.2014.0801)
Supplement: Supplementary material [file rsbl20140801supp1.pdf]

# Supplementary material

## 1) Solving equation (2.1) for adult mortality

Our aim is to solve $\mu$ from equation (2.1):

$$\frac{\sigma f e^{-rb}\left(1-e^{-c(r+\mu)}\right)}{r+\mu} = 1 \qquad (2.1)$$

To simplify this, we use the shorthand notation $y = \sigma f c e^{-rb}$ and $x = c(r + \mu)$. Equation (2.1) then simplifies to

$$\frac{y(1-e^{-x})}{x} = 1 \qquad (A1)$$

If we can now solve *x* from (A1), we can consequently easily solve $\mu$. (A1) contains both *x* and the exponential of *x*, which suggests the solution may involve the Lambert W function (Corless et al. 1996). Therefore we first need to rearrange (A1) to a form equivalent with $f(x)e^{f(x)} = g(y)$, where the right side does not contain *x*. This can be done with the following steps:

$$\frac{y(1-e^{-x})}{x} = 1$$ (Multiply both sides by *x* and rearrange)

$$x - y = -ye^{-x}$$ (Multiply both sides by $e^{(x-y)}$)

$$(x-y)e^{(x-y)} = -ye^{-y}$$ (This is of the form $f(x)e^{f(x)} = g(y)$. Now apply the Lambert W function)

$$(x-y) = W(-ye^{-y})$$ (Solve for x)

$$x = y + W(-ye^{-y})$$

Given that $x = c(r + \mu)$, it is now easy to solve $\mu$:

$\mu = \frac{x}{c} - r = \frac{y + W(-ye^{-y})}{c} - r$ , which is equation (2.2b).

The argument $-ye^{-y}$ is always negative. For negative arguments there are two possible real-valued branches to the Lambert W function. The correct branch can be determined by examining the left side of the equation $(x - y) = W(-ye^{-y})$. We will show that in biologically relevant scenarios the values of $(x - y)$ always fall in the interval ]-1,0[, indicating that we must use the principal branch of the Lambert W function (Corless et al. 1996):

From equation (A1) we get $y = \frac{x}{1-e^{-x}}$. Therefore

$(x - y) = x - \frac{x}{1-e^{-x}} = \frac{x}{1-e^{x}}$. It is easy to show that for positive values of *x*, the values of $\frac{x}{1-e^{x}}$ always fall in the interval ]-1,0[, and therefore the same applies to $(x - y)$. Noting that $x = c(r + \mu)$ is always positive in biologically relevant scenarios, we have shown that

$$\mu = \frac{y + W(-ye^{-y})}{c} - r \qquad (2.2b)$$

where *W* indicates the principal branch of the Lambert W function.

**Corless, R. M., G. H. Gonnet, D. E. Hare, D. J. Jeffrey, and D. E. Knuth. 1996. On the Lambert W function. Advances in Computational mathematics 5:329-359.**

**2) Continuous mortality increase**

For mathematical tractability, we derived equations (2.3a-d) with the assumption of a truncated form of aging: adult individuals have a constant mortality rate, until they reach age *d* after which they no longer reproduce (i.e. in the absence of extrinsic mortality, adults survive or reproduce for exactly *c* time units after maturation). Here we compare the results to numerical solutions derived with the continuous Gompertz-Makeham mortality distribution (e.g. Marshall and Olkin 2007), which is commonly used to model senescence.

With this distribution analytical equivalents of equations (2.3a-d) are not possible, but with numerical methods we can plot an equivalent of figure 1C, which allows us to compare the effect of the two alternative distributions.

To make the results comparable, we need a way to parameterize the Gompertz-Makeham distribution so that it reflects the value of *d*, as used in the main text. It is not immediately clear what the equivalent of this parameter is in a continuous distribution, because a continuous distribution does not have an absolute maximum age. One way to draw a parallel between the two is to note that *in the absence of extrinsic mortality*, all new recruits survive for a further *c*=*d*-*b* time units when using the truncated distribution. An equivalent continuous distribution would be one where, in the absence of extrinsic mortality, new recruits survive *on average* a further *c*=*d*-*b* time units after maturation.

The mortality rate at age *x* with the Gompertz-Makeham distribution is $\mu + \beta\varphi e^{\varphi x}$, where $\mu$ is an age-independent (extrinsic) mortality component, similar to the main text, while $\beta$ and $\varphi$ parameterize the age-specific component of mortality. The corresponding survival function is $e^{-(\beta(e^{\varphi x}-1)-\mu x)}$.

The mean of the age-specific (i.e. exluding $\mu$) component of the distribution is $\frac{e^{\beta}\Gamma(0,\beta)}{\varphi}$, where $\Gamma(0,\beta)$ is the incomplete gamma function. This is what we want to equate with *c*=*d*-*b* from the main model (because we are interested in survival after maturation). Therefore we proceed as follows: assign $\beta$ a value (we will use $\beta$=0.05 for the example shown here), then solve $\frac{e^{\beta}\Gamma(0,\beta)}{\varphi} = c$ for $\varphi$, which yields $\varphi = \frac{e^{\beta}\Gamma(0,\beta)}{c}$. Then numerically solve the equivalent of equation (2.2b) (in the case of *r*=0) from the main text, i.e. solve the required value of age-dependent adult mortality $\mu$ that yields a stable population. We will then have all the parameters required to calculate genetic generation time. These steps can be done with Mathematica by defining the following functions:

```
beta = 0.05;

phi[b_, d_]: = Exp[beta] Gamma[0, beta]/(d − b) ;

surv[u_, b_, d_, x_]: = Exp[−beta(Exp[phi[b, d](x − b)] − 1) − u(x − b)];

u[b_, f_, d_]: = u/. FindRoot[NIntegrate[f surv[u, b, d, x], {x, b, Infinity}] == 1, {u, 1}];

gt[b_, f_, d_]: = {utemp = u[b, f, d]; NIntegrate[x f surv[utemp, b, d, x], {x, b, Infinity}]};
```

The first four lines generate a continuous survival distribution analogous with the truncated one, and the final function calculates genetic generation time. With this we can plot the equivalent of figure 1C using the Gompertz-Makeham mortality distribution.

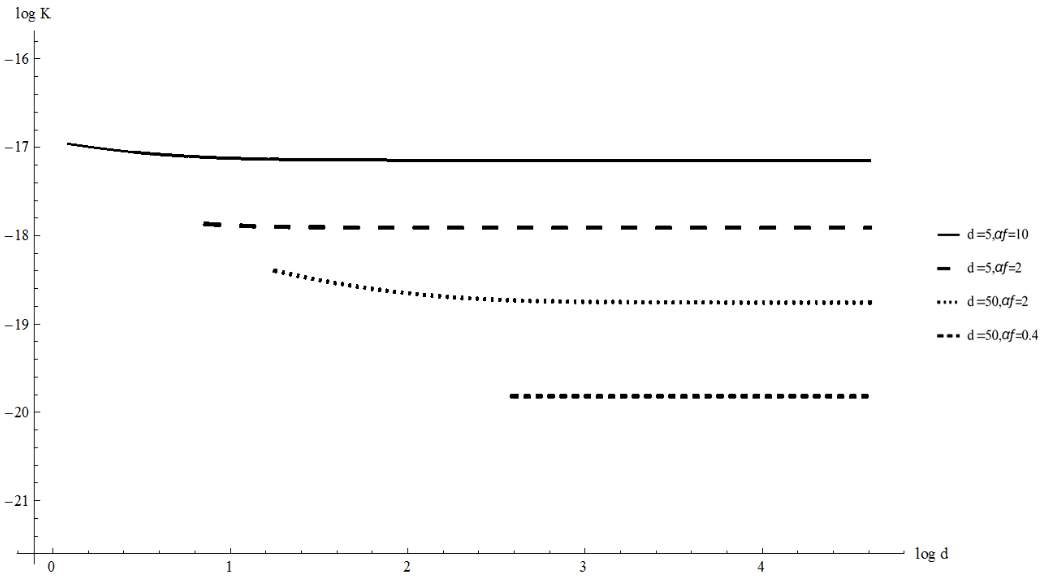


The figure is almost indistinguishable from figure 1C. This demonstrates that our main result is not an artefact of the truncated mortality function used in the main text.

For comparison, the dashed line below shows the age-dependent (intrinsic) component of the survival function used in the main text compared to the age-dependent component of the survival function used in this appendix, both with a mean survival of 20 years. Using the functions defined above, the command for the continuous distribution below is $\mathrm{surv}[0,0,20,x]$ Note that decreasing the value of $\beta$ from 0.05 would make the continuous distribution gradually approach the stepwise one.

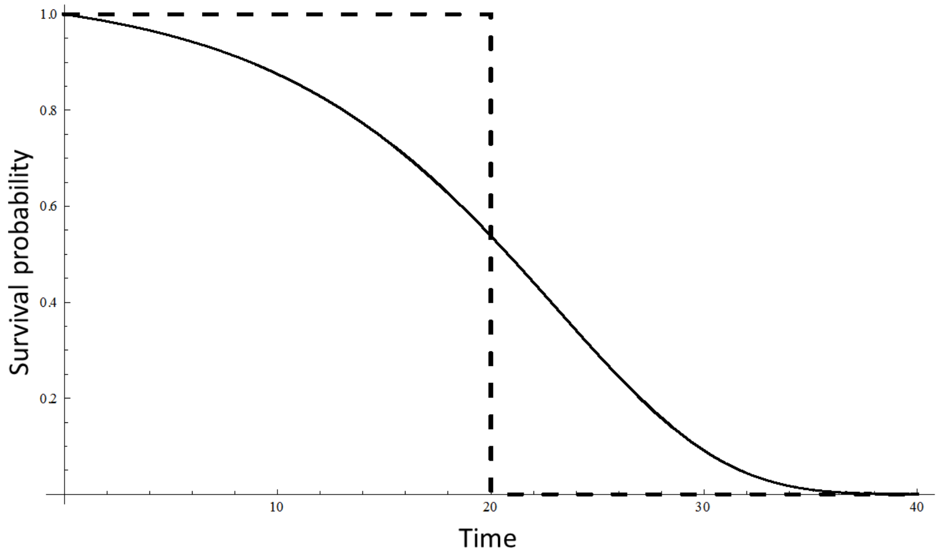



**Marshall, A. W. and I. Olkin. 2007. Life Distributions: Structure of Nonparametric, Semiparametric, and Parametric Families. Springer.**

**3) General fecundity and mortality functions**

Assume we are comparing species where the general shape of the life-history functions is very similar, varying only in their relative recruitment rate $\sigma f$ and an extrinsic mortality (after age of first reproduction) $\mu$ that balances recruitment so the population is in demographic equilibrium. The baseline fecundity and mortality functions can now take any age-specific shape, while $\sigma f$ and $\mu$ are simply scaling factors (but not independent of each other under demographic equilibrium).

We can write the survival and fecundity functions as $l(x) = \sigma e^{-\mu(x-b)}m(x)$ and $f(x) = fn(x)$, where *m*(*x*) and *n*(*x*) determine the overall shape of survival and fecundity, and can be any suitable functions.

Then the Euler-Lotka equation for *r*=0 (no population growth) yields

$$\int_b^\infty \sigma e^{-\mu(x-b)}m(x)fn(x)dx = 1$$

Or, using the notation *k*(*x*)=*m*(*x*)*n*(*x*),

$$\int_b^\infty \sigma f e^{-\mu(x-b)}k(x)dx = 1$$

Differentiating both sides for recruitment $\sigma f$, we get

$$\int_b^\infty e^{-\mu(x-b)}k(x)dx - \int_b^\infty \sigma f(x-b)\frac{\partial\mu}{\partial\sigma f}e^{-\mu(x-b)}k(x)dx = 0$$

or

$$\frac{1}{\sigma f}\int_b^\infty \sigma f e^{-\mu(x-b)}k(x)dx - \frac{\partial\mu}{\partial\sigma f}\int_b^\infty \sigma f x e^{-\mu(x-b)}k(x)dx + \frac{\partial\mu}{\partial\sigma f}b\int_b^\infty \sigma f e^{-\mu(x-b)}k(x)dx = 0$$

And using the Euler-Lotka and generation time equations to simplify:

$$\frac{1}{\sigma f} - \frac{\partial\mu}{\partial\sigma f}T + \frac{\partial\mu}{\partial\sigma f}b = 0$$

Now we can solve for *T*:

$$T = b + \frac{1}{\sigma f\frac{\partial\mu}{\partial\sigma f}} \qquad \text{(A2)}$$

The only difference between (A2) and equation (2.4) $T = b + \frac{1}{\sigma f}$ is the factor $\frac{\partial\mu}{\partial\sigma f}$ in the denominator. Therefore, whatever the overall shape of age-specific survival and fecundity, we still expect there to be a saturating relationship between *K* and recruitment rate, but the details might differ (encapsulated by $\frac{\partial\mu}{\partial\sigma f}$). For the special case of constant adult mortality and fecundity with no senescence, mortality must equal recruitment under demographic equilibrium. This implies that $\frac{\partial\mu}{\partial\sigma f} = 1$, i.e. equation (2.4) is recovered. Equation (2.3a) (with *r*=0) in the main text is an example where $\frac{\partial\mu}{\partial\sigma f} \neq 1$, and an additional factor is required.
